# The Effects of BCDs in Unilateral Conductive Hearing Loss: A Systematic Review

**DOI:** 10.3390/jcm12185901

**Published:** 2023-09-11

**Authors:** Xin-Yue Wang, Liu-Jie Ren, You-Zhou Xie, Yao-Yao Fu, Ya-Ying Zhu, Chen-Long Li, Tian-Yu Zhang

**Affiliations:** 1 Department of Facial Plastic Reconstructive Surgery, ENT Institute, Eye and ENT Hospital, Fudan University, Shanghai 200031, China; 17301050239@fudan.edu.cn (X.-Y.W.); renliujie@fudan.edu.cn (L.-J.R.); entxyz@fudan.edu.cn (Y.-Z.X.); fuyaoyao2007@126.com (Y.-Y.F.); yayingz@163.com (Y.-Y.Z.); 2 NHC Key Laboratory of Hearing Medicine, Fudan University, Shanghai 200031, China

**Keywords:** bone conduction devices, auditory benefits, unilateral conduction hearing loss, systematic review

## Abstract

Bone conduction devices (BCDs) are widely used in the treatment of conductive hearing loss (CHL), but their applications on unilateral CHL (UCHL) patients remain controversial. To evaluate the effects of BCDs in UCHL, a systematic search was undertaken until May 2023 following the PRISMA guidelines. Among the 391 references, 21 studies met the inclusion criteria and were ultimately selected for review. Data on hearing thresholds, speech recognition, sound localization, and subjective questionnaire outcomes were collected and summarized. Moderate hearing threshold improvements were found in UCHL patients aided with BCDs. Their speech recognition abilities improved significantly. However, sound localization results showed wide individual variations. According to subjective questionnaires, BCDs had an overall positive influence on the daily life of UCHL patients, although several unfavorable experiences were reported by some of them. We concluded that the positive audiological benefits and subjective questionnaire results have made BCDs a credible intervention for UCHL patients. Before final implantations, UCHL patients should first go through a period of time when they were fitted with non-implantable BCDs as a trial.

## 1. Introduction

Conductive hearing loss (CHL) occurs when sound waves cannot reach the inner ear due to defects of sound pathways in the outer and/or middle ear. Patients with unilateral conductive hearing loss (UCHL), i.e., CHL in one ear, suffer from hearing problems caused by the head shadow effect and sound localization troubles. 

To rehabilitate the binaural sound processing in UCHL patients, reconstructive ear surgery or middle ear implants are usually recommended. However, when surgeries are not feasible, and a traditional hearing aid is not acceptable, bone conduction devices (BCDs) become a favorable alternative. BCDs utilize the bone conduction pathway (bypasses the normal air-conduction pathway that involves the outer and middle ear) to reconstruct auditory perception and, thus, are very effective for the treatment of CHL. BCDs are classified into non-implantable and implantable devices. Non-implantable BCDs require no surgical interventions, e.g., the non-invasive ADHEAR devices (MED-EL, Innsbruck, Austria) can simply stick to the skin [1]. Implantable BCDs could provide higher auditory benefits. Since the first implantation (BAHA) in the late 1970s [2], various types of implantable devices have emerged, including the percutaneous BCDs (e.g., Baha Connect and the Ponto^®^ (Oticon Medical, Copenhagen, Denmark)), transcutaneous passive BCDs (e.g., Baha^®^ Attract system (Cochlear, Sydney, Australia) and the Sophono device (Sophono Inc., Boulder, CO, USA)) and transcutaneous active BCDs with an implanted actuator (e.g., Bonebridge^®^ (MED-EL, Innsbruck, Austria), Osia^®^ (Cochlear, Sydney, Australia)) [3]. BCDs are successful in curing bilateral CHLs, such as bilateral microtia and atresia. However, the applications of BCDs in UCHL patients remain controversial, partly because of the limited clinical data. In this review, we aim to evaluate the efficacy of BCDs in the treatment of UCHL, by collecting and analyzing the current research data on the applications of BCDs to UCHL.

## 2. Methods

### 2.1. Search Strategy

A systemic search was undertaken until May 2023. PubMed and Web of Science databases were the two major databases we searched. The search terms were: PubMed database: (((((BCHA[Title/Abstract]) OR (BCHAs[Title/Abstract])) OR (bone conduction hearing[Title/Abstract])) OR (bone conduction hearing aids[Title/Abstract])) OR (BAHA[Title/Abstract])) AND (conductive hearing loss, unilateral[MeSH Terms] OR (unilateral conductive hearing loss[Title/Abstract])). Web of Science (all data base): ((TS = (unilateral hearing loss)) OR TS = (single sided deafness)) AND (TS = (BCHAs) OR TS = (BAHA)). This study is conducted according to the Preferred Reporting Items for Systematic Reviews and Meta-Analyses (PRISMA) [4] and the Cochrane Handbook for Systematic Reviews of Interventions [5].

### 2.2. Inclusion and Exclusion Criteria

Studies were selected for this review if they conform to the following criteria: (1) the study subjects were patients with unilateral conductive or mixed hearing loss (2) the study subjects received bone-conduction hearing aids (BCHAs)/BCDs as intervention (3) the study reported outcomes of audiological measures and/or questionnaires (4) the study design was retrospective or prospective. Studies were particularly excluded if they: (1) Only included SSD or other unilateral sensorineural hearing loss, (2) Only included patients with bilateral hearing loss, (3) Used air conduction or cartilage conduction hearing aids, (4) Did not meet the inclusion criteria. There was no restriction on language. After primary search, duplicates were removed before the title and abstract review was undertaken. The studies were selected based on the inclusion/exclusion criteria.

### 2.3. Data Collection and Analysis

Two independent reviewers extracted the information separately. The included study types were all clinical trials, either retrospective or prospective. Before final inclusion, all the studies underwent quality assessments using the Agency for Healthcare Research and Quality (AHRQ) scale. Disagreements on study selection or quality assessment were resolved through a full discussion with a third reviewer. The outcomes of interest were hearing thresholds, speech recognition, sound localization, and subjective questionnaires. Other data points of interest included: study design, number and mean age of subjects, etiology of deafness, and types of BCDs. In studies that also examined non-criterion-meeting subjects (e.g., subjects with bilateral hearing loss, and subjects with SSD), we only collected information on the UCHL subjects.

## 3. Results

### 3.1. General Characteristics of the Studies

**Figure 1:**
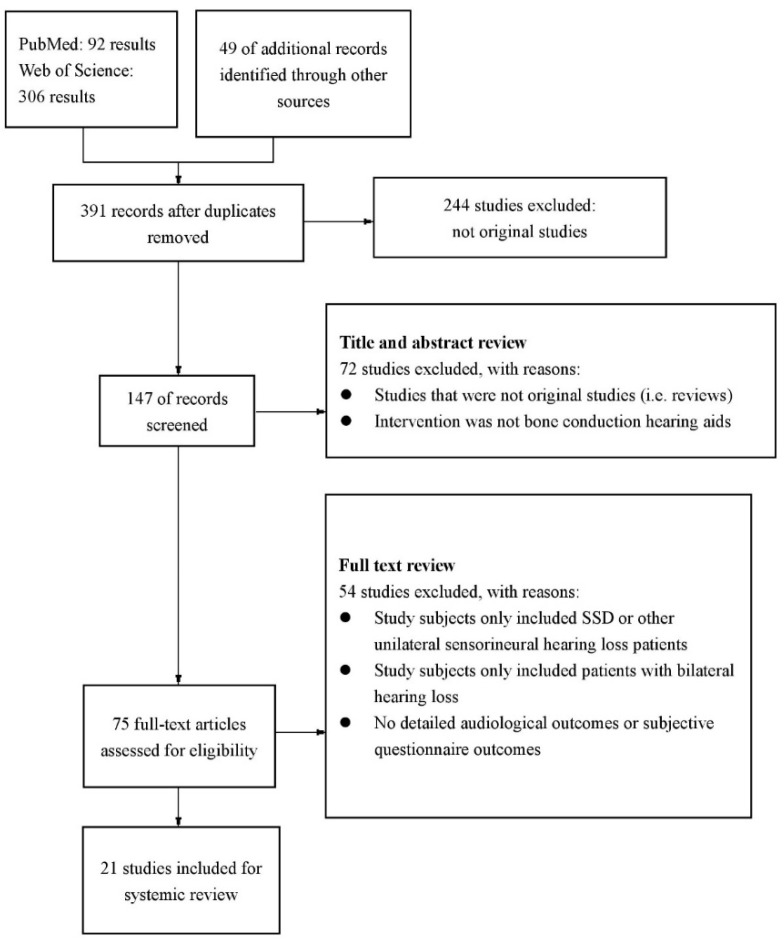
Study design: inclusion and exclusion of searching results.

The primary search yielded 398 results, an additional 49 studies were hand-searched and added. Fifty-six duplicates were removed, and 244 studies were removed for not being original studies. Afterwards, 147 studies remained for title and abstract review, and 72 studies were excluded. Then we full-text reviewed the rest 75 studies, and 54 of them were excluded based on the inclusion and exclusion criteria. Ultimately, 21 studies were decided suitable to be included in this systemic review (see Figure 1). Table 1 summarizes the study characteristics of the 21 studies we analyzed.

In all of the studies, the unaided and aided conditions were compared through prospective or retrospective analysis. Three of the studies recruited normal-hearing as control groups [6,7,8], whilst the majority of the studies were controlled before and after the study. Some studies included different patient groups [8,9,10,11]. Several studies analyzed both unilateral and bilateral CHL patients [9,10,12,13]. One study compared UCHL patients with normal bilateral cochlear function and those with mild symmetrical sensorineural hearing loss [14]. Some of the studies also intended to compare the effects of different BCDs. One compared ADHEAR with Ponto on softband aids [13], and one compared BoneBridge with Vibrant SoundBridge (VSB) [7], one compared ADHEAR with BAHA5 on softband [15].

**Table 1:** Study characteristics.

Study (Year)	Study Design	Patient Characteristics			Bone-Conduction Hearing Device
		Mean Age at Time of Study (Range)	N	Type of Unilateral Hearing Loss	
Brotto (2023) [16]	prospective	9 (6–11)	10	conductive	BAHA
Luque (2023) [17]	retrospective	10 (5–17)	9	conductive	Baha Attract system
Marszał (2022) [10]	prospective	41.1 (22–50)	7	mixed or conductive	Baha Attract system
Cywka (2021) [9]	prospective	1.2 (0.4–1.6)	21	mixed or conductive	Softband BCHA
Kuthubutheen (2020) [15]	prospective	40.3 (11–70)	12	conductive	BAHA; ADHEAR
de Wolf (2011) [11]	retrospective	9 (5–16)	15	conductive	BAHA
Kunst (2008) [18,19]	prospective	17.25 (5–61)	20	conductive	BAHA
Priwin (2007) [8]	prospective	9.4 (6–17)	13	conductive	BAHA
Hol (2005) [14]	prospective	43.2 (16–66)	18	conductive	BAHA
Snik (2002) [20]	prospective	39.4 (19–51)	8	conductive	BAHA
Wazen (2001) [21]	prospective	45 (23–76)	9	mixed or conductive	BAHA
Nelissen (2016) [22]	retrospective	7.8 (5–11)	12	conductive	Sophono and BAHA
Polonenko (2016) [23]	retrospective	12.1 (5–17)	9	conductive	Sophono
Denoyelle (2015) [24]	prospective	8.1 (5.1–10.8)	15	conductive	Sophono
Vogt (2018) [7]	prospective	11.3 (3.5–17.9)	9	conductive	BoneBridge
Vyskocil (2017) [25]	prospective	35.2 (14–50)	5	conductive	BoneBridge
Liu (2022) [6]	prospective	7.45 (5–11)	11	conductive	ADHEAR
Liu (2021) [26]	retrospective	7.8 (5–15)	13	conductive	ADHEAR
Hirth (2021) [27]	prospective	7 (4.0–16.7)	10	conductive	ADHEAR
Osborne (2019) [13]	prospective	9 (5–15)	20	conductive	ADHEAR and Ponto

The studies were done in the UK [13], USA [21], Canada [17,23], China [6,26], Germany [27], the Netherlands [7,11,14,18,19,20,22], Sweden [8], Australia [15], France [24,28], Italy [16], Poland [9,10], and Austria [25]. Patients of all ages are included in these studies, and both congenital and acquired UCHL are included. Causes of UCHL mainly include congenital unilateral microtia and atresia (CUMA), congenital ossicular chain anomaly, ear canal stenosis, mastoidectomies secondary to chronic ear infections, cholesteatoma, and temporal bone tumor excised.

The BCDs used are Bone-anchored hearing aids (BAHA) [8,11,14,18,20,21], Sophono [22,23,24], Baha Attract system [10,17], BoneBridge [7,25], as well as the ADHEAR hearing system [6,13,15,26,27].

### 3.2. Audiological Outcomes

Sound field hearing thresholds (pure tone average (PTA) gain), speech recognition (sometimes described as speech discrimination or speech perception), and sound localization test results are valuable audiological outcomes that reflect speech-identification and localization abilities. Only 16 out of the 21 included studies presented these audiological outcomes. Here we collected the available audiological data of the studies and summarized the outcomes in Table 2.

#### 3.2.1. PTA and Sound Field Hearing Threshold Gain

Of the 21 studies included in our analysis, 10 tested pure tone audiograms and sound field hearing thresholds. Overall, the aided hearing thresholds show great improvement when compared with unaided. For sound field hearing threshold tests, most studies had speakers placed 1 m distance in front of the subject’s head. Warble tones at frequencies of 0.5, 1, 2, and 4 kHz were presented. With the normal ear blocked, the mean tone thresholds of the above frequencies, i.e., the PTA, were usually measured. When aided with BAHA, Wazen et al. [21] reported the average gain (difference of preoperative and postoperative aided thresholds) was substantial at each of these frequencies. The greatest gain (66.3 dB) was obtained at 1000 Hz. When aided with Baha^®^ Attract, Brotto et al. [16] reported a PTA improvement from 63.2 ± 6.9 to 12.6 ± 4.7 dB (50.8 dB gain), and Luque et al. [17] reported a PTA gain of 39.9 dB. For studies that applied Sophono devices, the PTA of the impaired ear significantly improved from 69.1 ± 11.6 to 20.4 ± 8.5 dB HL [23], and from 69.02 ± 9.31 dB to 33.49 ± 4.89 dB HL [24], 58 dB to 31 dB HL [22], respectively. In studies that chose ADHEAR as the intervention, the hearing thresholds improved from 55 ± 2.4 dB HL to 31 ± 7.9 dB HL [13], 51.9 ± 4.9 dB HL to 26.5 ± 4.3 dB HL [26], and 53.9 dB HL to 26.4 dB HL [15], 51.36 ± 5.02 dB HL to 27.64 ± 2.38 dB HL [6], respectively, with all improvements being statistically significant. As for BoneBridge, the patients’ average hearing gain was 25.7 ± 2.6 dB [25].

#### 3.2.2. Speech Recognition

Of the 21 studies, 10 investigated speech recognition (some described as speech discrimination or speech perception) in quiet and in noise. The setups for testing speech recognition were similar to that for testing sound field hearing thresholds, except that speech signals were played. Speech tests were consistent with the subjects’ native language. Speech reception threshold (SRT), speech-to-noise ratio (SNR) at which the patient score was 50% (SRT_50_) and word recognition score (WRS) were used to represent the speech recognition ability. A decrease in SNR represents an improvement in speech recognition ability. Some studies tested noise from various angles, while some studies tested with one fixed noise speaker. Generally, speech recognition in quiet improved in at least a portion of the subjects tested [15,18,20,21,23,27]. When noise was presented on the impaired ear side, theoretically, deterioration might be observed because the BCDs were all fitted to the impaired ear side, which would eliminate the positive effect of head shadow. However, Snik et al. [20] observed no significant deterioration in any of the patients. Other studies [14,18] reported wide interindividual variation. Liu et al. reported slight improvements with no statistical significance [26]. When noise was presented from the contralateral ear side, theoretically, there should be a significant improvement. Most studies [14,18,20,26] reported outcomes that met this expectation, whilst others [23] reported limited improvements. When noise was presented in the front, great improvements were reported. Priwin et al. discovered that SRT improved in more noisy conditions [8]. Polonenko et al. found all except 1 subject improved or performed similarly when the noise came from the front and explained this result with binaural summation [23]. Kuthubutheen et al. [15] reported a mean unaided SNR of 2.23 dB that improved to 0.64-0.91 dB for ADHEAR or other BCDs. Liu et al. found an improvement of 2.5 ± 1.6 dB SNR after subjects were fitted with ADHEAR [26]. Vyskocil et al. reported a mean unaided SRT of 11.5 dB and an aided SRT of 2.9 dB [25].

#### 3.2.3. Sound Localization

Under normal hearing conditions, comparisons of the sounds reaching each ear are critical for accurate sound localization and, thus, depriving hearing on one side should degrade spatial hearing. Theoretically, rehabilitating binaural hearing by using BCDs might improve sound localization abilities.

In most study setups, several loudspeakers were placed in a circle with a radius of 1–1.5 m. The average angle difference between the actual sound-emitting speaker and the speaker indicated by the patient, or mean absolute error (MAE) is calculated as an outcome in 5 of the studies [7,14,19,20,22]. The perfect MAE is 0, and a decrease in MAE represents a positive outcome [19]. Root-mean-square (RMS) is another common calculation to describe localization accuracy [29]. The study of Vyskocil et al. [25] calculated the RMS. In addition, one study [22] used a minimum audible angle (MAA) test [30] to examine the localization accuracy.

General improvements in sound localization were found in five studies [7,14,20,22,25]. However, three studies reported cases of insignificant improvements and deteriorations [6,8,19]. The detailed statistics are summarized in Table 2. The evident difference in sound localization outcomes between congenital UCHL and acquired UCHL groups was highlighted in several studies. More detailed information about this phenomenon can be found in the Section 4 of this review.

### 3.3. Subjective Outcomes

Various scales were developed to evaluate the auditory behavioral, and daily-use information of different BCDs. 15 of the 21 studies used subjective questionnaires to evaluate the behavioral and developmental outcomes of UCHL patients. The detailed information of the studies was reviewed and summarized in Table 3.

#### 3.3.1. APHAB

Abbreviated Profile of Hearing-Aid Benefit (APHAB) [31] is a 24-item questionnaire, consisting of four 6-item subscales: Ease of Conversation (EC), Reverberation (RV), Background Noise address speech understanding (BN), and Aversiveness of sounds (AV). Of the 11 studies involved, 3 studies [10,11,23] used the APHAB questionnaire. Two of the studies reported ambiguous outcomes [11,23], while the remaining study reported significant improvements with a mean gain of 38.6% (*p* < 0.001) [10]. The outcomes show wide variations and cannot make a solid conclusion possibly because of the insufficient sample size of the patient groups.

#### 3.3.2. GBI&GCBI

The Glasgow Benefit Inventory (GBI) [32,33] and the Glasgow Children’s Benefit Inventory (GCBI) [17,34] were used in four of the studies [10,11,13,19]. The response to each question is calculated to provide a mean total score that ranges from −100 (maximum deterioration) to +100 (maximum improvement). An overall benefit of +34 [19], 26 ± 22 [11], 33 ± 25 [13], +14.6 [17] and 20.6 ± 18.6 [10] demonstrated positive feedbacks of the BCD users.

#### 3.3.3. SSQ

The Speech, Spatial, and Qualities of Hearing Scale (SSQ) [35] aims to measure a range of hearing abilities across several domains. It contained three domains of hearing ability: speech perception, spatial hearing, and quality of hearing. Kunst et al. [19] focused on the spatial hearing domain. On a scale from 0 to 10, the mean score increased from 4.5 to 6.8 (*p* = 0.046) for adults aided with BAHA. In the children’s version of SSQ, the group showed a total mean score of 6.6 with the BAHA. Hirth et al. [27] also reported a significant increase from 6.5 ± 1.8 to 7.8 ± 1.0 (*p* = 0.0313). Kuthubutheen et al. [15] reported an increase from 73 to 83.9 with BAHA and 90.1 with ADHEAR in the SSQ12 questionnaire, which was significant (*p* < 0.05). Liu et al. [6] also reported a significant increase in each subdomain.

#### 3.3.4. IOI-HA

IOI-HA refers to the International Outcome Inventory for Hearing Aids. Seven items are included: use, benefit, residual activity limitation, satisfaction, residual participation, impact on others, and quality of life. Scores above three indicate the success of the hearing aid fitting compared to the unaided situation. All of the studies that involved IOI-HA presented a mean score above three in all seven items [6,8,26].

#### 3.3.5. ADHEAR Use and Satisfaction Questionnaire

A specific 20-question subjective questionnaire for ADHEAR users was created to assess the effectiveness and ease of use of the device in subjects’ daily life during the testing period. Liu et al. retrospectively collected the subjective satisfaction of 13 subjects with UCHL who received ADHEAR, and they reported an average daily use time of 7.2 h, and 85% of them considered ADHEAR a valuable hearing aid in total [26]. Kuthubutheen et al. prospectively investigated 12 UCHL subjects, most of whom gave positive comments on the hearing device and reported a mean daily use time of 8.9 h [15].

#### 3.3.6. MAIS&MUSS

Two studies [8,24] contained a meaningful auditory integration scale and meaningful use of speech scale (MAIS&MUSS) questionnaire [36]. Their results were conflicting. Priwin et al. [8] reported wide variation in problems with hearing aid function and spontaneous hearing aid use in children with UCHL. The patients had almost unchanged scores in unaided and aided situations, and the aided verbal communication score even lowered. Denoyelle et al. [24] reported that all children used the device 5 to 12 h daily without cutaneous complications 12 months after BCD implantation, with both children and parents being satisfied or very satisfied. The score for 7/10 questions in silence or in a noisy environment was statistically improved.

#### 3.3.7. Other Subjective Questionnaire Outcomes

Studies that involved the Chung and Stephens’ questionnaire [37] reported overall satisfaction and long daily-use time of BAHA [14,19]. Significant benefits were reported in one study that selected a 10 cm Linear Analogue Scale (LAS) to measure the subjective health status perceived by UCHL patients when aided and unaided with ADHEAR [13]. As to the Bern Beneft in Single-Sided Deafness questionnaire(BBSS) [38], no negative effect was reported except for one response to the second question (speech in noise) by 1 patient, who reported hearing whistling from his device in noisy environments [26]. Hearing Handicap Inventory for Adults (HHIA) [39] also revealed a novel reduction in handicaps after BAHA implants [21]. The 15-item Satisfaction with Amplification in Daily Life (SADL) [40] used in the study by Polonenko et al. [23] indicated overall “considerable” satisfaction with the device. A special scale named LittlEARS was used to measure the auditory development situation in one special study that investigated 42 children under 2 years old with congenital microtia and atresia [9]. Half of the subjects had bilateral CHL, the rest had UCHL. The researchers concluded that the average LittlEARS score increased significantly after the children were provided with BCDs. Notably, the bilateral CHL group had a larger increase (average difference = 15.33, *p* < 0.001) in comparison to the UCHL group (average difference = 5.91, *p* < 0.001).

## 4. Discussion

### 4.1. Auditory Benefits and Wide Variations

Differences were found between congenital UCHL and acquired UCHL patients, especially in sound localization tests. Subjects with congenital UCHL somehow already have good test results unaided [18,20,41], therefore, their aided results showed little improvement or even deterioration. In the study of Agterberg et al. [41], the researchers also revealed that there were huge differences in congenital and acquired UCHL patients after being fitted with BCDs. MAE score of the congenital UCHL group only changed minorly from 34 ± 24 to 30 ± 13, while the acquired UCHL group improved significantly from 46 ± 20 to 12 ± 10 (*p* < 0.025). This result also supported the opinions stated in the studies of Hol et al. [14], Kunst et al. [18], and Snik et al. [20]. The well-localization abilities in congenital UCHL patients might be an outcome of adaptation over the years without hearing aids. According to neurological studies, adaptation to asymmetric hearing loss can either be accomplished by reinterpreting altered spatial cues or by relying more on intact cues. Adaptation of monaural deprivation is also possible in adulthood [42], but with less flexibility. This adaptation cannot fully compensate for the deficiency in hearing abilities, since both groups still perform worse than the normal hearing group.

For studies that included both unilateral and bilateral CHL groups, the audiological beneficial effects were much more significant in the bilateral CHL groups. This was reasonable because the UCHL patients did not experience as much inconvenience as bilateral CHL patients before BCD treatments. In the case of UCHL patients, the improvement of living quality and hearing abilities in real-life situations seemed to be of more importance. To decide whether BCDs provide UCHL patients with actual audiological benefits, unmasked hearing thresholds are very valuable indicators. The study of Polonenko et al. [23] revealed that speech perception abilities in noise only subtly improved while unmasked, whilst significant improvements in the implanted ear were found when masking the contralateral ear in quiet conditions. The auditory benefits received by UCHL patients were limited, compared to bilateral CHL groups. In the UCHL groups, large individual differences were found.

### 4.2. Subjective Outcomes

Twelve different types of questionnaires were involved in the selected studies. Only a few studies adopted the same questionnaire and therefore the sample size was relatively small. In spite of this, questionnaire outcomes showed that patients have an overall high satisfaction rate and optimistic attitudes towards the BCDs.

BCD applications come with the potential cost of convenience, comfort, money, and appearance. These are the aspects we should value more when applying BCDs and receiving feedback from patients.

### 4.3. Nuts and Bolts of Different BCDs

Comparative works on audiological and subjective outcomes among different BCDs were also available in some of the literature. Hol et al. [14], Denoyelle et al. [24], and Nelisson et al. [22] compared BAHA(percutaneous BCD) with Sophono(transcutaneous BCD). Sophono Alpha1 demonstrated non-inferiority compared to BAHA1 on a test-band, with its good cutaneous tolerance, satisfaction of users, and improvement of the quality of life in one study [24]. When it comes to skin reactions, despite case reports about postoperative soft tissue complications after BAHA implantation [43,44], new implant designs have been created and proved sufficient to minimize skin reactions [45,46]. In comparative studies that measured audiological outcomes, however, the Sophono groups did not achieve as much hearing improvement as the BAHA groups [22,47]. The BoneBridge (BB, MED EL) is a relatively new subcutaneously implanted bone conduction implant with an implantable portion and an external audio processor. Fewer complications were reported in BB users because the BB leaves the skin intact. Although the implantation age was once limited by bone thickness, the latest study proved that the second generation (BCI 602), which features a decreased implant thickness with a reduced surgical drilling depth can be implanted safely in young children with good postoperative hearing performance [48]. The ADHEAR system also has its own merits and limits. Despite the fact that the ADHEAR provided lower sound amplification, a previous study has shown that ADHEAR worn for longer than headband-worn BCHA, has a high user satisfaction rate while causing no skin pain or irritation [49]. When it comes to the influence of daily life, an ideal BCD should have a comfortable wearing experience and an inconspicuous appearance, and most importantly, it should meet the patients’ daily requirements to minimize communicative limitations and localize sounds. Therefore, the ADHEAR device could be a good choice for UCHL patients if they find the sound amplification level is sufficient for their daily use.

### 4.4. Advices for Clinical Intervention

Children with unilateral hearing loss often have worse language and speech performance than their peers [50]. Children with UCHL usually do not show much inconvenience in life than those with unilateral sensorineural hearing loss (SNHL), possibly because they still have two normal functioning cochleae and a normal bone conduction hearing pathway. Although children with UCHL tend to have better school performance than those with SNHL, most of them still report communication and behavioral problems and need some sort of resource assistance [51,52]. Recently, a neuroimaging study has revealed abnormally high brain activities in the left inferior temporal gyrus of UCHL patients, which is positively associated with the duration of hearing loss [53]. This finding demonstrated that even partial hearing deprivations, such as UCHL can cause progressive alterations in functional brain networks. Additionally, evidence showed that chronic CHL leads to cochlear degeneration, and the olivocochlear efferent pathway has dramatic use-dependent plasticity even in the adult ear [54]. Therefore, eliminating audiometric asymmetry is not only necessary to the children’s early speech-language development and auditory cortex development but also important in adulthood. BCDs can provide binaural cues for patients with congenital UCHL, thus assisting the development of horizontal plane localization abilities [25] and avoiding future disturbance of voice communication [9]. Furthermore, even if surgery for congenital aural atresia is performed successfully, audiological results suggest a more consistent hearing outcome with bone-anchored hearing aids [55,56]. Thus, our advice on the most favorable time for BCD use is as follows: to guarantee early speech-language development, BCDs should be fitted in congenital UCHL patients as soon as hearing screening results indicate UCHL. As for acquired UCHL patients who already have adequate language development, BCDs could be applied if needed. However, it is important that they go through “in vivo” trials with non-implantable devices before the final decision. The time of BCD use may influence the central auditory reconstruction of acquired UCHL patients, but there is currently no solid evidence on the best time point to apply the BCDs for them.

Due to the wide individual differences of UCHL patients when fitted with BCDs, we highly recommend that all UCHL patients who wish to use implantable BCDs should first go through a period of time when they were fitted with non-implantable BCDs as a trial. If the audiological benefits and satisfaction were promising, then implantations could be carried out successively.

## 5. Conclusions

BCDs deliver an overall benefit to patients with UCHL. It is important that UCHL patients receive BCDs to rehabilitate binaural hearing. Children with congenital UCHL should be treated early to reach proper speech-language development and auditory cortex development. Adults with UCHL can also use BCDs to aid their hearing and localizing abilities. Due to the wide individual variations, we recommend a period of trials on headband BCDs to help decide whether long-term usage or implantations are necessary. The type of BCD should be selected according to individual conditions.

## Figures and Tables

**Table 2:** Audiological outcomes.

Study	Measures	Main Outcomes
Wazen (2001) [21]	PTA gain	500-Hz: 41.9 dB 1000-Hz: 66.3 dB 2000-Hz: 49.4 dB 4000-Hz: 45.6 dB
Snik (2002) [20]	Sound localization	MAE (significant change ≥ 16°) ● 2000-Hz&500-Hz noise ■ 5/8 significant improvement (5 acquired UCHL) ■ 3/8 already low MAE unaided (2 congenital UCHL, 1 acquired UCHL)
	Speech recognition	SRT (Significant improvement at 5% level) ● in quiet ■ 4/8 significant improvement ● speech in front and the noise on the normal side ■ 7/8 significant improvement ● speech in front and the noise on the impaired side: ■ no significant deterioration or improvement
Hol (2005) [14]	Sound localization	Patients with normal bilateral cochlear function (all with acquired UCHL) MAE (significant change ≥ 16°) ● 500-Hz noise ■ 5/13 significant improvement ■ Average MAE improvement of 18° ● 3000-Hz noise ■ 7/13 significant improvement ■ Average MAE improvement of 18° Patients with mild symmetrical sensorineural hearing loss MAE (significant change ≥ 16°) ● 500-Hz noise ■ 3/5 significant improvement ■ Average MAE improvement of 43° ● 3000-Hz noise ■ 2/5 significant improvement ■ 2/5 significant deterioration ■ Average MAE improvement of 22°
	Speech recognition	Patients with normal bilateral cochlear function SRT (Significant improvement at 5% level) ● in quiet: ■ 9/13 significant improvement ● speech in front and the noise on the normal side: ■ 11/13 significant improvement ● speech in front and the noise on the impaired side: ■ no significant deterioration or improvement Patients with mild symmetrical sensorineural hearing loss SRT (Significant improvement at 5% level) ● in quiet: ■ 4/5 significant improvement ● speech in front and the noise on the normal side: ■ 2/5 significant improvement ● speech in front and the noise on the impaired side: ■ 1/5 significant deterioration ■ 2/5 significant improvement
Kunst (2008) [18]	Sound localization	(all with congenital UCHL) Adults: mean unaided MAE score = 37° (significant change ≥ 16°) ● 500-Hz noise ■ 3/10 significant improvement ■ 2/10 significant deterioration ● 3000-Hz noise ■ 2/10 significant improvement ■ No significant deterioration Children: mean unaided MAE score = 54° ● 500-Hz condition (significant change ≥ 27°) ■ 3/10 significant improvement ■ 2/10 significant deterioration ● 3000-Hz condition (significant change ≥ 34°) ■ 2/10 significant improvement ■ No significant deterioration
	Speech recognition	Adults: SRT (improvement of >1.6 dB was significant) ● In quiet: ■ 4/10 significant improvement ● speech in front and the noise on the normal side: ■ 4/10 significant improvement On average, the change in S/N ratio with the BAHA was 0.4 dB, which was not statistically significantly different from zero. Children: SRT (improvement of >1.6 dB was significant) ● speech in front and the noise on the normal side: ■ 5/8 significant improvement ■ 23% more phonemes repeated correctly
Denoyelle (2015) [24]	PTA gain	Mean aided ACPTA of 33.49 ± 4.89 dB; mean gain 35.53 dB (*p* < 0.0001) at M6 Mean aided ACPTA of 36.43 ± 4.61 dB at M12
Nelissen (2016) [22]	Sound localization	MAA test (all with congenital UCHL) ■ Sophono group ◆ Mean Unaided 52°; aided14° ■ BAHA group ◆ Unaided 80°; aided23°
Polonenko (2016) [23]	Speech recognition	mean aided SRT 51.40 ± 10.99; SRT gain (−7.80 ± 4.11) (*p* < 0.001) at M6 mean aided SRT of 39 ± 5.86 dB at M12
Vyskocil (2017) [25]	Functional hearing gain Speech recognition Sound localization	Average: 25.7 dB (±2.6) ● WRS score median improvement 60% points (55–70). ● SRT_noise_ median improvement S_0_N_0_: 11.1 dB (7.5–12.4 dB) S_90_N_−90_: 9.0 dB (8.8–11.4 dB) mean RMS error decreased by a factor of 0.71 (*p* < 0.001)
Vogt (2018) [7]	Sound localization	MAE: mean unaided MAE = 35.5° Stimuli at the impaired side: improvement by 17° (*p* = 0.02) Stimuli at the normal side: no significant improvement or deterioration
Osborne (2019) [13]	PTA gain Sound field hearing gain	31 dB HL 26.3 dB HL
Kuthubutheen (2020) [15]	PTA gain Speech recognition	27.5 dB HL unaided SNR = 2.23 aided SNR = 0.64
Liu (2021) [26]	Sound field hearing gain Speech recognition	500-Hz: 24.2 ± 6.4 dB HL 1000-Hz: 27.7 ± 7.8 dB HL 2000-Hz: 26.9 ± 10.5 dB HL 4000-Hz: 22.7 ± 8.1 dB HL ● WRS gain ■ in quiet: 1.9 ± 2.5%, *p* < 0.05 ■ in noise: 7.3 ± 5.3%, *p* < 0.001 ● SNR gain ■ S_0_N_0_: 2.5 ± 1.6 dB SNR (*p* < 0.001) ■ S_0_N_NH_: 2.9 ± 1.6 dB SNR (*p* < 0.001) ■ S_MA_N_NH_: 5.7 ± 3.4 dB SNR (*p* < 0.001) ■ S_0_N_MA_: no significant difference
Hirth (2021) [27]	Functional hearing gain Speech recognition	19.6 dB HL (*p* < 0.0039) ● WRS improvement compared to unaided situation in quiet: 50.5% (*n* = 10, *p* < 0.0039) in noise: 35% (*n* = 6, *p* < 0.0313) ● SRT_50_ in quiet: Unaided average threshold: 69.4 ± 8.0 dB SPL signifcantly improved by 16.6 dB SPL to 52.8 ± 7.1 dB SPL using the hearing device (*n* = 9, *p* < 0.0195) ● SRT50 in noise: 0.2 ± 5.5 dB SNR unaided −1.7 ± 3.9 dB SNR aided
	Functional hearing gain Speech recognition	23.73 ± 3.47 dB HL (*p* < 0.01) ● WRS in quiet: ■ unaided 18.27 ± 14.63% ■ aided: 85.45 ± 7.38 % (*p* < 0.01) ● SRT in noise: ■ unaided: −5 ±1.18 dB SPL ■ aided: −7.73 ± 1.42 dB SPL (*p* < 0.05)
Liu (2022) [6]	Sound localization	(all with congenital UCHL) ● mean MAE on impaired side: ■ unaided: 43.18 ± 30.58° ■ aided: 34.14 ± 17.9° (no significant improvement) ● mean MAE on normal side: ■ unaided: 26.97 ± 24.68° ■ aided: 27.42 ± 14.53° (no significant improvement)
Luque (2023) [17]	PTA gain	39.9 dB
Brotto (2023) [16]	PTA gain	50.6 dB HL
	Speech recognition	● SIMT in noise: S_0_N_0+180_ median SNR unaided = −5.6 dB HL S_0_N_0+180_ median SNR aided = −6.4 dB HL (significant improvement, *p* = 0.027)

Abbreviations: MAA = minimum audible angle; MAE = mean absolute error; PTA = pure tone average; RMS = root mean square; SNR = speech-to-noise ratio; SRT_50_ = speech reception threshold at which each patient scored 50%; S_0_N_0_ = speech signal (S) and noise (N) were presented from the front; S_0_N_NH_ = speech signal was from the front with the noise from the healthy side; S_MA_N_NH_ = speech signal was from the CUMA (MA) side with the noise from the normal hearing (NH) side; S_0_N_MA_ = speech signal from the front with noise towards the atretic ear; S_0_N_0+180_ = speech signal from the front and noise in front and behind the subject; WRS = Word recognition scores; SIMT = Italian Matrix Sentence Test.

**Table 3:** Subjective outcomes.

Study	Questionnaire	Main Outcomes
Wazen (2001) [21]	HHIA	Preoperative: mean score = 25 (range, 10–40), falling in the “moderate” handicapped range. Postoperative: mean score = 10 (range, 0–14), falling in the borderline of “mild to moderate” handicap perception range. The reduction in handicap for the individual data for these unilaterally impaired listeners was dramatic.
Hol (2005) [14]	Chung and Stephens’ questionnaire	The majority of patients were using their BAHA 7 days a week for more than 8 h a day. The majority of the patients prefer using BAHA when listening to speech in both quiet and noisy situations.
Priwin (2007) [8]	MAIS&MUSS	Rare to occasional hearing aid use were reported in the UHL group. The aided and unaided scores was almost unchanged, and the aided verbal communication score even lowered.
	IOI-HA	In the UCL with single sided BAHA amplification group, mean score all 7 items ≥3. High satisfaction rate and high quality of life were reported after fitted with hearing amplification.
Kunst (2008) [19]	Chung and Stephens’ questionnaire	Most of the patients gave preference to using the BAHA system in several everyday situations. When asked whether they would recommend the BAHA to another patient with same hearing disability, all the patients gave a positive response.
	GCBI	Overall benefit of +34 (children *n* = 10)
	SSQ	Unaided 4.5, aided 6.8
de Wolf (2011) [11]	Daily use	47% were using their BAHA devices for more than 8 h a day, and 40% were using them for 4 to 8 h a day
	GCBI	Total score 26 (mean = 22); physical health 16(mean = 19)
	APHAB	27% experienced a significant overall benefit (scores of 10 + for each subdomain)
	HUI-3	The overall mean utility score was 0.82 (0.12)
Denoyelle (2015) [24]	MAIS&MUSS	At M12, all children used the implant 5 to 12 h daily (mean: 10 h) without cutaneous complications. Both children and parents reported being satisfied or very satisfied. The score for 7/10 questions in silence or noisy environment was statistically improved when wearing the device
Polonenko (2016) [23]	APHAB	only 3/8 children had minor changes in all three subscales and therefore significant overall benefit. All except two children reported a major change in at least one subscale, mainly background noise and reverberation
	SADL	Median ratings of satisfaction (global score = 5.0, positive effect = 5.3, service and cost = 6.5, negative features = 5.0, personal image = 4.7) did not significantly differ from 5, or “considerably satisfied” for all subscales (*p* > 0.05), indicating adequate satisfaction with the device.
Osborne (2019) [13]	LAS	The mean LAS score increased by 4.5 from 4 ± 1.4 to 8.5 ± 1.4 *p* = 0.0001 (95% CI 5.23–3.53)
	GCBI	Overall GCBI response scores increased following the use of the adhesive retained BC system for 4 weeks by33 ± 25, further analysis shows a positive score in all four dimensions.
Kuthubutheen (2020) [15]	SSQ12	The mean unaided SSQ score was 73 which significantly improved to 83.9 with the BCHA device and 90.1 with the ADHEAR
	ADHEAR Use and Satisfaction Questionnaire	Daily use: 5–14 h (mean: 8.9 h) Most patients considered the device ”valuable”.
Liu (2021) [26]	IOI-HA	The mean score of the IOI-HA was 4.0 ± 0.5 without any negative comments
	BBSS	the total score of all 10 questions was 27.1 ± 10.1
	ADHEAR use and satisfaction questionnaire	The hearing device provided benefits in speech recognition ability in different complex situations, with high satisfaction rates.
Hirth (2021) [27]	SSQ	significant increase from 6.5 ± 1.8 to 7.8 ± 1.0 (*p* = 0.0313)
Cywka (2021) [9]	LittlEARS	the average score increased significantly from pre-treatment period. The average difference of UHL group was 5.91 (*p* < 0.001; *e*^2^ = 0.264)
Marszał (2022) [10]	GBI	total score:20.6 ± 18.6 (*p* = 0.026), improvement general scale: 35.7 ± 28.7 points (*p* = 0.016), improvement physical health subscale: −14.3± 31.1 (*p* = 0.270) deterioration
	APHAB	mean gain = 34.0% (*p* = 0.008)
Liu (2022) [6]	IOI-HA	mean overall IOI-HA score = 4.57 ± 0.73
	SSQ	significant increase from 6.33 ± 1.82 to 8.37 ± 1.05 (*p* < 0.01)
Luque (2023) [17]	GCBI	the median GCBI score was +14.6, indicating overall positive benefit 89% patients had an overall quality of life benefit largest improvement was found in behavior subscale

Abbreviations: GBI = Glasgow Benefit Inventory; GCBI = Glasgow Children’s Benefit Inventory; APHAB = Abbreviated Profile of Hearing-Aid Benefit; SSQ = Speech, Spatial and Qualities of hearing scale; SADL = Satisfaction with Amplification in Daily Life; LAS = 10 cm Linear Analogue Scale; HHIA = the Hearing Handicap Inventory for Adults; IOI-HA = International outcome inventory for hearing aids; BBSS = The Bern benefit in single-sided deafness questionnaire; MAIS&MUSS = meaningful auditory integration scale and meaningful use of speech scale.

## Data Availability

Data can be provided for academic purposes on request from the primary author.
